# Abietane and nor-abitane diterpenoids from the roots of *Salvia rhytidea*

**DOI:** 10.1186/s40064-016-2652-0

**Published:** 2016-07-13

**Authors:** Farrokh Eghtesadi, Mahdi Moridi Farimani, Nourallah Hazeri, Jafar Valizadeh

**Affiliations:** Department of Chemistry, Faculty of Science, University of Sistan and Baluchestan, Zahedan, P.O. box 98135-674, Iran; Department of Phytochemistry, Medicinal Plants and Drugs Research Institute, Shahid Beheshti University, G. C., Evin, Tehran, Iran; Department of Biology, Faculty of Science, University of Sistan and Baluchestan, Zahedan, P.O. box 98135-674, Iran

**Keywords:** *Salvia rhytidea*, Diterpenoid, Abietane, Solvent extraction, Column chromatography, Structure elucidation

## Abstract

**Background:**

The genus *Salvia* is a rich source of structurally diverse terpenoids. Different species of the *Salvia* have been used in folk medicine of Iran and therefore attracted the attention of researchers for exploring their chemical constituents. In a project directed at structurally interesting bioactive metabolites from Iranian *Lamiaceae*, we studied *Salvia rhytidea*.

**Results:**

Fractionation of the petroleum ether extract of the root of *S. rhytidea* led to the isolation of a new 20-nor-abietane diterpenoid (**1**), together with seven known compounds, comprising five abietane diterpenoids (**2**–**6**), and two rearranged abietanes (**7**, **8**). Their structures were established by a combination of 1D and 2D NMR.

**Conclusions:**

Our results showed that the root of *S. rhytidea* could be considered as a new and rich source of different types of abietane and rearranged abietane diterpenoids.

## Background

The genus *Salvia* is a rich source of structurally diverse terpenoids (Kintzios 2000; Moridi Farimani et al. 2013). Among these, numerous diterpenoids with promising bioactivities, such as antileishmanial, antitumor, antimicrobial, antifungal properties, have been reported from *Salvia* species (Ebrahimi et al. 2013; Tan et al. 2002; Akaberi et al. 2015; Moridi Farimani and Miran 2014; Ulubelen 2003; Jassbi et al. 2006). The most abundant diterpenoids in the genus are abietanes and rearranged abietanes (Wu et al. 2012). The genus *Salvia* is represented in the Iranian flora by 61 species, of which 17 are endemic (Jamzad et al. 2012). *Salvia rhytidea* Benth is an endemic species that grows widely in the eastern parts of Iran (Rechinger 1987). In our efforts to discover new and potentially bioactive secondary metabolites from Iranian *Salvia* species (Moridi Farimani and Mazarei 2014; Ebrahimi et al. 2014; Moridi Farimani et al. 2012; Bahadori et al. 2015; Moridi Farimani et al. 2008), we investigated the petroleum ether extract of the root of *S. rhytidea*. Here we report the isolation and structure elucidation of 1-deoxo aurocadiol (**1**), as a new 20-nor-abietane diterpenoid. In addition, the abietane diterpenoids ferruginol (**2**), taxodione (**3**), arucadiol (**4**), deoxyneocryptotanshinone (**5**), and 7α- Ethoxyroyleanone (**6**), and rearranged abietanes microstegiol (**7**) and 12-hydroxysapriparaquinone (**8**) were isolated and are described here for *S.rhytidea* for the first time.

## Results and discussion

**Fig. 1:**
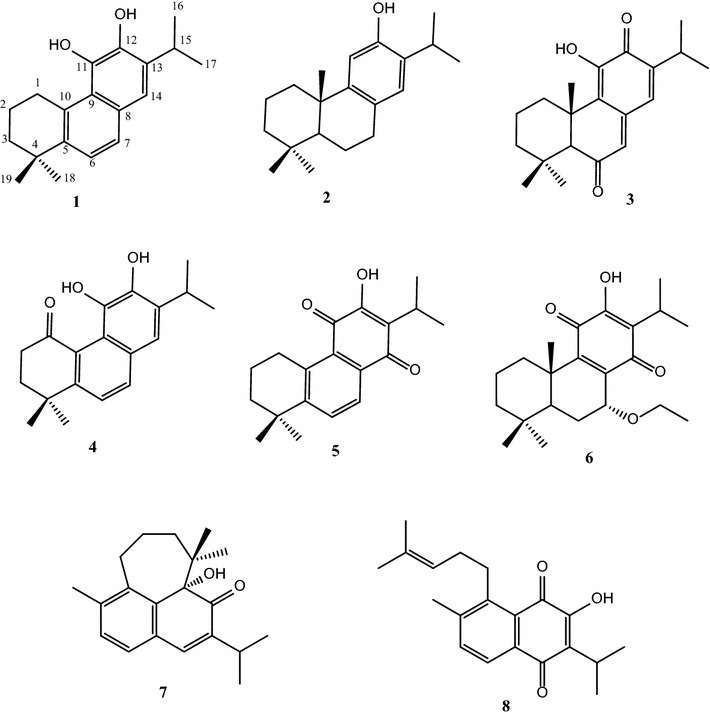
Structure of compounds **1**–**8**

**Fig. 2:**
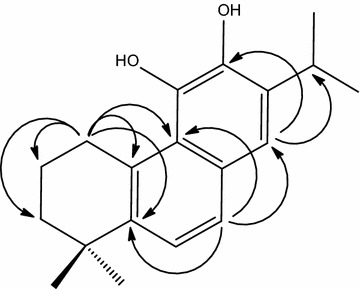
Key HMBC correlations of **1**

Compound **1** (Fig. 1) was isolated as an orange, amorphous solid. The IR spectrum showed absorptions of hydroxy (3475 cm^−1^) and olefinic (1610 cm^−1^) functionalities. The ^13^C NMR spectrum showed 19 carbon resonances, which were identified with the aid of HSQC and DEPTQ spectra as four methyl, three methylene, four methine, and eight quaternary carbons. The ^13^C NMR spectrum showed signals indicative of ten aromatic carbons. The ^1^H NMR spectrum showed resonances of two methyl singlets at *δ*_H_ 1.22 (s, 6H). Resonances of two additional methyl groups at *δ*_H_ 1.14 (d, *J* = 6.9 Hz) and 1.19 (d, *J* = 6.9 Hz), together with a signal at *δ*_H_ 2.90 (sept, *J* = 6.9 Hz) indicated the presence of an isopropyl moiety. Signals at *δ*_H_ 7.02 (d, *J* = 7.0 Hz) and 7.43 (d, *J* = 7.0 Hz) were indicative of two aromatic protons with *ortho*-position to each other. Another aromatic methine signal appeared as a singlet at *δ*_H_ 6.78. Therefore, the structural features were reminiscent of a nor-abietane diterpenoid containing two aromatic rings. The NMR data of **1** showed great similarity to those of arucadiol isolated from *S. argentea* (Michavila et al. 1986) and *S. miltiorrhiza* (Ginda et al. 1988). Inspection of the ^13^C NMR spectra showed the lack of a carbonyl group in compound **1** but the presence of an additional methylene group at *δ*_C_ 27.6. The chemical shifts of C-2, C-5, and C-7 were observed at *δ*_C_ 18.5, 148.8, and 126.5, respectively, with upfield shifts of ca. 18, 10, and 11 ppm relative to arucadiol, while the chemical shift of C-3, C-6, C-9, and C-10 were observed at *δ*_C_ 38.0, 129.8, 128.4, and 134.6 with downfield shift of ca. 3, 10, 9, and 10 ppm, respectively. These observations suggested the replacement of the C-1 carbonyl with a methylene group. HMBC correlations (Fig. 2) between H-1 (*δ*_H_ 2.78, 2H, *t*), and C-2 (*δ*_C_ 18.5), C-5 (*δ*_C_ 148.8), C-10 (*δ*_C_ 134.6), and C-9 (*δ*_C_ 128.4), and COSY correlation between H-1 and H-2 (*δ*_H_ 1.72, 2H, m) confirmed the location of the methylene group. Unambiguous assignments of NMR data were achieved by a combination of COSY, HMQC, and HMBC experiments. Compound **1** was therefore established structurally as 1-deoxo-arucadiol.

Ferruginol (**2**) is a well known abietane diterpenoid which was isolated from different *Salvia* species such as *S. syriaca* and *S. sclarea* (Ulubelen et al. 2000, 1994). Antileishmanial (Tan et al. 2002), antimicrobial (Ulubelen et al. 1999), cytotoxic (Moujir et al. 1996; Fronza et al. 2011), antihypertensive (Ulubelen et al. 1994), and anticholinesterase (Topcu et al. 2013) activities were reported for ferruginol and the mechanism of its antioxidant properties was also investigated (Saijo et al. 2015).

Taxodione (**3**) is a diterpenoid with quinone methide skeleton which was reported from different genus like *Taxudium*, *Clerodendrum*, and *Salvia* (Machumi et al. 2010; Kolak et al. 2009; Kusumoto et al. 2009). Different biological properties have been reported for this compound, including antibacterial (Yang et al. 2001), antioxidant (Kolak et al. 2009), antitermitic (Kusumoto et al. 2009), antifeedant (Acosta et al. 2008), antifungal (Topçu and Gören 2007), and anticholinesterase activities (Topcu et al. 2013). Moreover, cytotoxic and tumor inhibitory properties of taxodione have been investigated in in situ and in vivo experiments (Kupchan et al. 1969; Ulubelen et al. 1999; Abou Dahab et al. 2007). The mechanism of action of taxodione for its cytotoxic properties was investigated in several articles with focus on its DNA binding and DNA damaging character (Zaghloul et al. 2008), and its enzyme inhibitory action (Hanson et al. 1970).

Arucadiol (miltiodiol) (**4**) was previously extracted from *S. argentea* (Michavila et al. 1986), *S. miltiorrhiza* (Ginda et al. 1988), *S. prionitis* (Yong 1995), and *S. apiana* (González et al. 1992). Cytotoxic properties have been reported for arucadiol (Fronza et al. 2011).

Deoxyneocryptotanshinone (**5**) is a *para*-quinone abitanne diterpenod with cytotoxic activity which was isolated from *S. miltiorrhiza* for the first time (Ikeshiro et al. 1991).

7α-Ethoxyroyleanone (**6**) has been reported from *S. lavandulaefolia, S. lanigra,* and *Peltodon longipes* and its cytotoxic and antioxidant effects were investigated (Fronza et al. 2011; Shaheen et al. 2011; Michavila et al. 1985; Burmistrova et al. 2013).

Microstegiol (**7**) is a rearranged abietane with a seven-member ring skeleton. This compound has solely been reported from *Salvia* genus so far (Topcu et al. 2013; Ulubelen et al. 1992). It has been shown to have mild antibacterial effects (Topçu and Gören 2007).

12-hydroxysapriparaquinone (**8**), a rearranged 4,5-seco-abietane diterpenoid, previously isolated from *S. limbata* (Topcu et al. 1996).

## Experimental

### General experimental procedures

NMR spectra were recorded at a target temperature of 18 °C on a Bruker Avance III 500 MHz spectrometer operating at 500.13 MHz for ^1^H and 125.77 MHz for ^13^C. 2D NMR experiments (^1^H-^1^H COSY, HSQC, HMBC, NOESY) were performed using Bruker microprograms. CDCl_3_ was purchased from Armar Chemicals. TLC was performed on silica gel (Merck, Kieselgel 60, F_254_, 0.25 mm) phase. Column chromatography (CC) was carried out using silica gel (70–230 mesh, Merck). Flash column chromatography (FCC) was performed on silica gel (230–400 mesh, Merck).

### Plant material

The roots of *Salvia rhytidea* were collected from Taftan Mountain, 28°36′ N and 61°4′ E, in the Baluchistan of Iran at an altitude of 2497 m, in autumn 2012. The plant was authenticated by Dr. Valizadeh and a voucher specimen (no. 4938) was deposited in the Herbarium of the School of Biology (Dr. Akhani Herbarium), University of Tehran.

### Extraction and purification

**Fig. 3:**
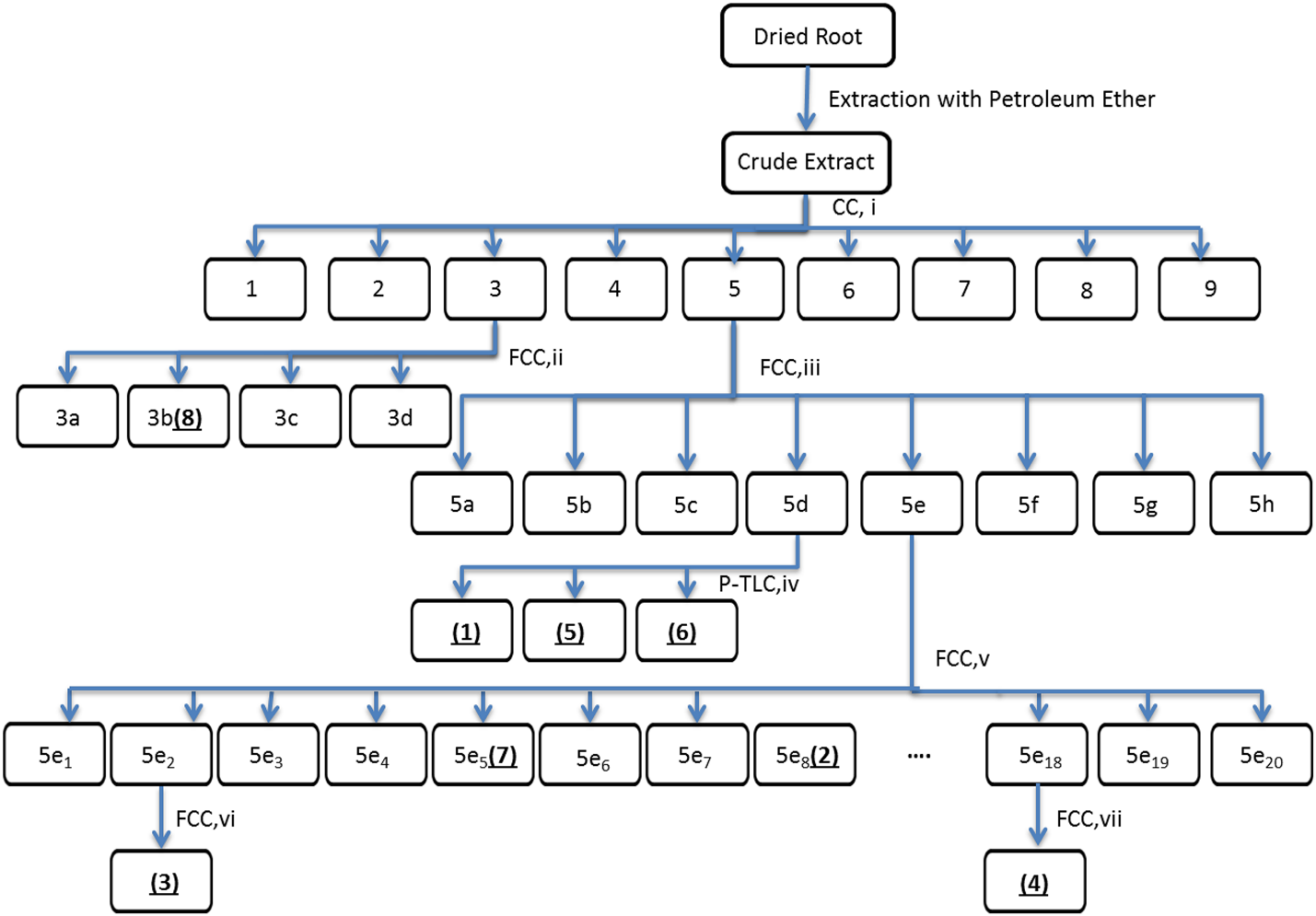
Schematic diagram of extraction and purification procedure for petroleum ether extract of *S. rhytidea* roots

The air-dried roots of *S. rhytidea* (2100 g) were crushed and extracted with petroleum ether (bp. 40–60 °C) at room temperature (25 °C) for 5 days. The extract was concentrated in vacuo, to afford 12.6 g dark gummy residue. It was subjected to a silica gel column chromatography using petroleum ether (3 L) as eluting solvent, increasing the polarity with a mixture of dichloromethane: acetone (90:10, 5 L), to yield nine fractions (1–9) (Fig. 3). Fraction 3 was subjected to silica gel flash column chromatography using petroleum ether-diethyl ether (98:2, 2.5 L), as mobile phase. Four subfractions (3a–3d) were collected. Subfraction 3b was recrystallized from Me_2_CO to afford **8** (8 mg). Fraction 5 was separated on a silica gel flash column with a gradient of petroleum ether-EtOAc (100:0, 0.5 L; 90:10, 0.3 L, 80:20, 0.3 L; 70:30, 0.3 L; 60:40, 0.2 L; 50:50, 0.2 L) as eluent, to yield 8 subfractions (5a–5h). Subfraction 5d was purified by preparative TLC [developed with acetone-EtOAc-petroleum ether (2:2:96)] to afford **1** (4 mg), **5** (5 mg), and **6** (5 mg). Subfraction 5e was separated over a silica gel flash column, eluting with petroleum ether-CH_2_Cl_2_ (85:15, 1 L) followed by increasing concentrations of CH_2_Cl_2_ (up to 100 %), to afford 20 subfractions (5e_1_–5e_20_). Compounds **7** (10 mg) and **2** (7 mg) were recrystallized from subfractions 5e_5_ and 5e_8_, respectively. Subfraction 5e_2_ was purified by another silica gel column to yield **3** (5 mg). Subfraction 5e_18_ was also separated on another silica gel column to yield **4** (3 mg).

### 1-Deoxoarucadiol (**1**)

^1^H-NMR (500 MHz, in CDCl_3_): δ 1.14 (3H, d, J = 6.9 Hz, Me-16), 1.19 (3H, d, J = 6.9 Hz, Me-17), 1.22 (6H, s, Me-18 and Me-19), 1.58 (2H, m, H-3), 1.72 (2H, m, H-2), 2.78 (2H, t, J = 6.3 Hz, H-1), 2.90 (1H, sept., J = 6.9 Hz, H-15), 6.78 (1H, s, H-14), 7.02 (1H, d, J = 7.0 Hz, H-7), 7.43 (1H, d, J = 7.0 Hz); ^13^C-NMR (125 MHz, in CDCl_3_): 18.5 (C-2), 20.6 (C-16 and C-17), 27.6 (C-1), 31.8 (C-18 and C-19), 32.4 (C-15), 34.0 (C-4), 38.0 (C-3), 126.5 (C-7), 128.4 (C-9), 129.8 (C-6), 131.6 (C-14), 134.6 (C-10), 139.7 (C-13), 140.4 (C-8), 148.8 (C-5), 152.9 (C-11), 161.2 (C-12).

### Ferruginol (**2**)

^1^H-NMR (500 MHz, in CDCl_3_); δ 0.92 and 0.95 (each 3H, s, Me-18 and Me-19), 1.17 (3H, s, Me-20), 1.22 (1H, m, H-3), 1.25 and 1.26 (each 3H, d, J = 7.0 Hz, Me-16 and Me-17), 1.33 (1H, d, J = 12.0 and 2.2 Hz, H-5), 1.37 (1H, m, H-1),1.48 (1H, m, H-3) 1.58 (1H, dt, J = 13.9 and 3.4 Hz, H-2), 1.68 (1H, brd, J = 12.9 Hz, H-6), 1.72 (1H, m, H-2), 1.88 (1H, tdd, J = 13.2, 7.4, and 1.8 Hz, H-6), 2.14 (1H, brd, J = 12.5 Hz, H-1), 2.80 (1H, dd, J = 11.4 and 7.4 Hz, H-7), 2.88 (1H, ddd, J = 11.4 and 7.4 Hz, H-7), 3.20 (1H, sept., J = 6.9 Hz, H-15), 5.34 (s, OH), 6.69 (1H, s, H-11), 6.86 (1H, s, H-14), ^13^C-NMR (125 MHz, in CDCl_3_): δ 20. 1(C-2), 20.4 (C-6), 22.7 (C-19), 23.7 (C-16), 23.8 (C-17), 26.2 (C-20), 27.7 (C-15), 31.0 (C-7), 33.4 (C-18), 34.5 (C-4), 38.8 (C-10), 40.0 (C-1), 42.8 (C-3), 51.5 (C-5), 111.8 (C-11), 127.5 (C-14), 127.8 (C-8), 132.5 (C-13), 149.5 (C-9), 151.8 (C- 12).

### Taxodione (**3**)

^1^H-NMR (500 MHz, in CDCl_3_): δ 1.18 and 1.21 (each 3H, d, J = 6.9 Hz, Me-16 and Me-17), 1.15, 1.30 and 1.30 (each 3H, s, Me-18, Me-19 and Me-20), 1.20 and 1.44 (1H, m, H-3), 1.67 and 1.86 (each 1H, m, H-2), 2.64 (1H, brs, H-5), 2.97 (1H, ddd, J = 10.7, 3.6 and 1.3 Hz, H-1), 3.10 (1H, dsept., J = 6.9 and 0.8 Hz, H-15), 6.26 (1H, s, H-7), 6.91 (1H, brs, H-14), 7.66 (s, 11-OH); ^13^C-NMR (125 MHz, in CDCl_3_): δ 19.3 (C-2), 22.7 (C-20), 22.7 (C-16), 22.8 (C-17), 22.8 (C-19), 28.1 (C- 15), 33.0 (C-4), 33.6 (C-18), 38.3 (C-1), 42.7 (C-3), 43.5 (C-10), 63.1 (C-5), 126.7 (C-9), 135.1 (C-7), 137.5 (C-14), 140.0 (C-8), 145.1 (C-11), 146.2 (C- 13), 182.7 (C-12), 201.7 (C-6).

### Arucadiol (**4**)

^1^H-NMR (500 MHz, in CDCl_3_); δ 1.37 (6H, d, J = 6.9 Hz Me-16 and Me-17), 1.47 (6H, s, Me-18 and Me-19), 2.09 (2H, t, J = 6.8 Hz, H-3), 2.94 (2H, t, J = 6.8 Hz, H-2), 3.47 (1H, sept., J = 6.9 Hz, H-15), 6.89 (brs, 11-OH), 7.28 (1H, s, H-14), 7.33 (1H, d, J = 8.6 Hz, H-6), 7.95 (1H, d, J = 8.6 Hz, H-7), 10.73 (s, 11-OH), ^13^C-NMR (125 MHz, in CDCl_3_): δ 22.0 (C-16 and C-17), 27.4 (C-15), 29.6 (C-18 and C-19), 35.5 (C-3), 36.2 (C-2), 118.5 (C-14), 120.0 (C-11), 120.1 (C-6), 126.7 (C-10), 127.7 (C-9), 128.4 (C-8), 136.6 (C-13), 137.8 (C-7), 148.2 (C-12), 158.3 (C-5), 204.3 (C-1).

### Deoxyneocryptotanshinone (**5**)

^1^H-NMR (500 MHz, in CDCl_3_): δ 1.29 (6H, d, J = 7.0 Hz, Me-16 and Me-17), 1.30 (6H, s, Me-18 and Me-19), 1.79 (4H, m, H-2 and H-3), 3.20 (2H, t, J = 6.4 Hz), 3.37 (1H, sept., J = 6.5 Hz, H-15), 7.71 (1H, d, J = 8.2 Hz, H-6), 7.97 (1H, d, J = 8.2 Hz, H-7), 7.82 (1H, s, 12-OH); ^13^C-NMR (125 MHz, in CDCl_3_): 19.2 (C-2), 19.9 (C-16 and C-17), 24.5 (C-15), 30.0 (C-1), 31.8 (C-18 and C-19), 37.8 (C-3), 123.8 (C-13), 125.1 (C-7), 126.5 (C-9), 132.8 (C-8), 133.4 (C-6), 140.7 (C-4), 152.5 (C-10), 153.3 (C-12), 183.4 (C-11), 184.6 (C-14).

### 7α-Ethoxyroyleanone (**6**)

^1^H-NMR (500 MHz, in CDCl_3_): δ 0.84 and 0.87 (each 3H, s, Me-18 and Me-19), 1.11 (3H, t, J = 5.9 Hz, Me-2′), 1.12 and 1.15 (each 3H, d, J = 7.1 Hz, Me-16 and Me-17), 1.13 (3H, s, Me-20), 1.13 (1H, overlap, H-1), 1.17 (1H, overlap, H-3), 1.29 (1H, m, H-6), 1.39 (1H, brd, J = 13.1 Hz, H-3), 1.47 (1H, dt, J = 14.0, 3.5 Hz, H-2), 1.57 (1H, d, J = 12.8 Hz, H-8), 1.64 (1H, m, H-2), 1.93 (1H, d, J = 14.1 Hz, H-14), 2.60 (1H, brd, J = 13.1 Hz, H-1), 3.10 (1H, sept., J = 7.1 Hz, H-15), 3.61 (2H, m, H-1′), 4.35 (1H, dd, J = 3.2 and 1.6 Hz, H-7); ^13^C-NMR (125 MHz, in CDCl_3_): 15.5 (C-2′), 18.5 (C-2), 18.7 (C-16 and C-17), 18.8 (C-20), 21.5 (C-19), 22.8 (C-6), 23.4 (C-15), 32.5 (C-18), 33.0 (C-4), 35.2 (C-1), 38.9 (C-10), 40.5 (C-5), 45.2 (C-5), 65.0 (C-1′), 68.6 (C-7), 124.8 (C-13), 141.2 (C-8), 146.9 (C-9), 150.2 (C-12), 184.0 (C-11), 186.1 (C-14).

### Microstegiol (**7**)

^1^H-NMR (500 MHz, in CDCl_3_): δ 0.80 and 0.81 (each 3H, s, Me-18 and Me-19), 1.17 and 1.22 (each 3H, d. J = 6.9 Hz, Me-16 and Me-17), 1.27 and 2.4 (each 1H, m, H-3), 1.42 and 1.69 (each 1H, m, H-2), 2.33 (3H, s, H-20), 2.80 (1H, ddd, J = 14.0, 6.2 and 2.3 Hz, H-1), 3.03 (1H, dsept, J = 6.7 and 0.7 Hz, H-15), 3.61 (1H, dt, J = 2.1 and 13.1 Hz, H-1), 4.53 (s, 11-OH), 6.91 and 7.07 (1H, d, J = 7.6 Hz, H-7 and H-6), 6.97 (1H, d, J = 0.5 Hz, H-14); ^13^C-NMR (125 MHz, in CDCl_3_): 21.1 (C-16), 21.4 (C-20), 21.5 (C-18), 22.1 (C-17), 23.5 (C-2), 26.9 (C-1), 27.1 (C-15), 28.0 (C-19), 39.0 (C-4), 42.9 (C-3), 84.3 (C-11), 126.7 (C-7), 129.1 (C-8), 130.1 (C-6), 137.3 (C-5), 139.3 (C-9), 140.4 (C-14), 141.0 (C-13), 143.3 (C-10), 206.18 (C-12).

### 12-hydroxysapriparaquinone (**8**)

^1^H-NMR(500 MHz, in CDCl_3_): δ 1.29 (6H, d, J = 7.0 Hz, Me-16 and Me-17), 1.60 and 1.70 (each 3H, s, Me-18 and Me-19), 1.65 (2H, m, H-3), 2.15 (2H, q, J = 8.2 Hz, H-2), 2.42 (3H, s, Me-20), 3.16 (2H, brt, J = 8.2 Hz, H-1), 3.37 (1H, sept., J = 6.5 Hz, H-15), 5.27 (1H, t, J = 6.9 Hz, H-3), 7.47 (1H, d, J = 7.9 Hz, H-6), 7.83 (1H, s, 12-OH), 7.93 (1H, J = 7.9 Hz, H-7); ^13^C-NMR (125 MHz, in CDCl_3_): 17.6 (C-19), 19.9 (C-18 and C-19), 20.4 (C-20), 24.6 (C-15), 27.1 (C-1), 30.3 (C-2), 38.5 (C-3), 124.7 (C-13), 125.5 (C-7), 126.2 (C-9), 132.8 (C-8), 136.3 (C-6), 143.3 (C-5), 144.4 (C-10), 145.6 (C-4), 153.3 (C-12), 183.4 (C-11), 184.5 (C-14).

## Conclusions

Our results showed that the root of *S. rhytidea* contained different types of abietane and rearranged abietane diterpenoids. Biological properties of some of these compounds have been reported previously. Accordingly, *S. rhytidea* could be considered as a new and rich source of natural agents for the treatment of cancer, malaria, and microbial strains.
